# Factors Associated with Receiving a Discharge Care Plan After Stroke in Australia: A Linked Registry Study

**DOI:** 10.31083/j.rcm2310328

**Published:** 2022-09-28

**Authors:** Emma Polhill, Monique F Kilkenny, Dominique A Cadilhac, Natasha A Lannin, Lachlan L Dalli, Tara Purvis, Nadine E Andrew, Amanda G Thrift, Vijaya Sundararajan, Muideen T Olaiya

**Affiliations:** ^1^Stroke and Ageing Research, Department of Medicine, School of Clinical Sciences at Monash Health, Monash University, Clayton, VIC 3168, Australia; ^2^Stroke Theme, The Florey Institute of Neuroscience and Mental Health, University of Melbourne, Heidelberg, VIC 3084, Australia; ^3^Department of Neuroscience, Central Clinical School, Monash University, Melbourne, VIC 3800, Australia; ^4^Alfred Health, Melbourne, VIC 3800, Australia; ^5^Department of Medicine, Peninsula Clinical School, Monash University, and National Centre for Healthy Ageing, Frankston, VIC 3199, Australia; ^6^Department of Medicine, St Vincent’s Hospital, University of Melbourne, Fitzroy, VIC 3065, Australia

**Keywords:** patient discharge, quality of care, stroke, Australia, data linkage

## Abstract

**Background:**

Discharge planning is recommended to optimise the transition from acute care to home for patients admitted with stroke. Despite this guideline recommendation, many patients do not receive a discharge care plan. Also, there is limited evidence on factors influencing the provision of discharge care plan post-stroke. We evaluated patient, clinical and system factors associated with receiving a care plan on discharge from hospital back to the community after stroke.

**Methods:**

This was an observational cohort study of patients with acute stroke who were discharged to the community between 2009–2013, using data from the Australian Stroke Clinical Registry linked to hospital administrative data. For this analysis, we used merged dataset containing information on patient demographics, clinical characteristics, and receipt of acute care processes. Multivariable logistic regression models were used to determine factors associated with receiving a discharge care plan.

**Results:**

Among 7812 eligible patients (39 hospitals, median age 73 years, 44.7% female, 56.9% ischaemic stroke), 47% received a care plan at discharge. The odds of receiving a discharge care plan increased over time (odds ratio [OR] 1.39 per year, 95% CI 1.37–1.48), and varied between hospitals. Factors associated with receiving a discharge care plan included greater socioeconomic position (OR 1.18, 95% CI 1.02–1.38), diagnosis of ischaemic stroke (OR 1.18, 95% CI 1.05–1.33), greater stroke severity (OR 1.15, 95% CI 1.01–1.31), or being discharged on antihypertensive medication (OR 3.07, 95% CI 2.69–3.50). In contrast, factors associated with a reduced odds of receiving a discharge care plan included being aged 85+ years (*vs*<85 years; OR 0.79, 95% CI 0.64–0.96), discharged on a weekend (OR 0.56, 95% CI 0.46–0.67), discharged to residential aged care (OR 0.48, 95% CI 0.39–0.60), or being treated in a large hospital (>300 beds; OR 0.30, 95% CI 0.10–0.92).

**Conclusions:**

Implementing practices to target people who are older, discharged to residential aged care, or discharged on a weekend may improve discharge planning and post-discharge care after stroke.

## 1. Introduction

Over two-thirds of survivors of stroke experience enduring physical, cognitive, or emotional disability after discharge from acute care [1]. With the majority of survivors being discharged from acute care to the community [2], patients and caregivers face many challenges throughout post-stroke recovery and rehabilitation. These include inadequate provision of information, unmet needs for equipment and support services, and poor mental health [2, 3, 4]. Consequently, overcoming barriers in the transition from hospital, and planning for ongoing management in the community is important to mitigate these challenges and the risk of adverse events post-discharge.

The provision of a comprehensive discharge care plan, that has been co-developed with the patient, after acute stroke or transient ischaemic attack (TIA) is recommended in the Australian clinical guidelines and standards [5]. However, nearly one third of patients in Australia do not receive a care plan at discharge [6, 7], compared to 96% of patients in the United Kingdom [8]. The poor adherence to discharge care planning guidelines in Australia is of particular concern, given that many Australian survivors of stroke report having unmet needs long-term after discharge [9, 10]. Moreover, many survivors are readmitted to the hospital within the first 12 months following stroke [11, 12], potentially as a result of inadequate management of risk factors [13] or suboptimal management of stroke related impairments and poor adherence to prevention medications post-discharge [14]. Implementation of a comprehensive discharge care plan has the potential to reduce these unmet needs and mitigate any adverse events in survivors of stroke [15].

An understanding of the factors influencing the receipt of discharge care plans in the acute stroke care setting is necessary to guide improvements to this aspect of recommended care. Prior research on factors associated with discharge care planning after acute stroke has been limited to the investigation of single or few factors [16, 17, 18, 19, 20, 21]. We aimed to comprehensively evaluate patient, clinical and system factors associated with receiving a discharge care plan after acute stroke/TIA in Australia.

## 2. Materials and Methods

### 2.1 Study Design, Setting and Participants

This was an observational cohort study of patients with acute stroke or TIA admitted to one of 39 hospitals that participated in the Australian Stroke Clinical Registry (AuSCR) between 2009 and 2013. These hospitals covered rural and metropolitan regions of the states of Queensland, New South Wales, Victoria and Western Australia. The AuSCR is a national clinical quality registry which prospectively collects data for the purposes of monitoring adherence to processes of care during for patients hospitalised with acute stroke/TIA [22]. Patient-level data from the AuSCR were linked with hospital admissions and emergency presentations data, as part of the Stroke123 project [23]. For the present analysis, we included AuSCR registrants who were aged ≥18 years, had no missing data on age, sex or type of stroke, and were discharged alive to the community (home or residential aged care) from acute care following stroke/TIA.

### 2.2 Stroke123 Linked Dataset

In the AuSCR, data are routinely collected on patient characteristics, acute stroke care quality indicators (e.g., care plan received at discharge), hospital outcomes (e.g., in-hospital death, discharge destination), and patient outcomes between 90 and 180 days after stroke/TIA (e.g., health-related quality of life). The hospital admissions datasets contained information on dates of admission and discharge, urgency of admission, socio-demographics (e.g., age, sex, residential postcode), healthcare funding source (public or private) and *International Statistical Classification of Diseases and Related Health Problems*, *Tenth Revision*, *Australian Modification* (ICD-10-AM) diagnosis codes. The emergency presentation datasets included sociodemographic data, date and time of presentation, urgency of presentation (including triage category) and the primary diagnosis.

### 2.3 Definition of Variables

Discharge care planning was defined as having documented evidence in the medical record that a patient, family member or caregiver, has received a plan that outlines care in the community post-discharge, developed in conjunction with a multi-disciplinary care team [24]. It is also recommended that a care plan contains information on: (1) risk factor modification, (2) community support services and contacts, (3) further rehabilitation or outpatient appointments, and (4) equipment needed for recovery [24].

Patient characteristics included sociodemographic information, e.g., age, sex and residential postcode. Socioeconomic position was derived from each patient’s postcode of residence using quintiles of the Index of Relative Socioeconomic Advantage and Disadvantage (IRSAD) [25]. For the present analysis, socioeconomic position was classified as either disadvantaged (quintiles 1–2) or advantaged (quintiles 3–5). Other patient-level clinical characteristics included type of stroke, comorbidities, stroke severity, and processes of care received. Type of stroke was based on the diagnosis assigned by clinicians in the AuSCR. Comorbidities were derived from admission and emergency presentation data using ICD-10-AM diagnosis codes recorded within a 5-year look-back period up until and including the time of the stroke/TIA. Data on comorbidities were used to calculate the Charlson Comorbidity Index (CCI), a measure of multimorbidity that is predictive of 1-year mortality [26]. A patient’s inability to walk independently on admission to hospital was used as a validated, surrogate measure of stroke severity [27].

System-level factors included hospital characteristics (e.g., hospital location, bed size) and quality improvement indicators (e.g., management in a stroke unit and discharge on antihypertensive medication). Similar to the approach used in previous studies involving the review of medical records [6, 7], a patient was assumed to have not received a process of care if data were missing for that process of care. Using the Accessibility and Remoteness Index of Australia (ARIA+) [28], hospitals were classified as either metropolitan (ARIA+ category 1) or regional (ARIA+ categories 2 or 3). The size of hospital (e.g., small or large) was delineated based on having 300 or more beds, using information provided by the AuSCR office.

## 3. Statistical Analyses

Differences between patients who did and did not receive a discharge care plan were compared using χ^2^ tests for categorical variables and Mann-Whitney U tests for nonparametric, continuous variables (normality of data determined using Shapiro-Wilk test).

Multilevel (hospital, patient) multivariable logistic regression models, built using a parsimonious approach, were used to determine the factors associated with receiving a discharge care plan. Variables that reached a significance level of ≤0.1 in univariable analyses were included in the regression model. The only exceptions were year of stroke/TIA, age, sex, type of stroke, ability to walk on admission and CCI, which were included priori. Standard techniques were used to check for multicollinearity between independent variables, and a condition index of <25 was considered acceptable. A standard two-tailed alpha value of <0.05 was used and results were reported as odds ratios (ORs) with corresponding 95% confidence intervals (CIs). Data were analysed using Stata SE 16.0 (StataCorp, College Station, Texas, USA).

## 4. Availability of Data

Due to ethical and legal restrictions, linked administrative data from this study cannot be shared. However, aggregated data outputs and coding that support the findings of this study are available from the corresponding author on reasonable request, following approval from the relevant data custodians. Final linked data were available for analysis in 2018, five years after initial applications for data linkage.

## 5. Results

**Fig. 1.**
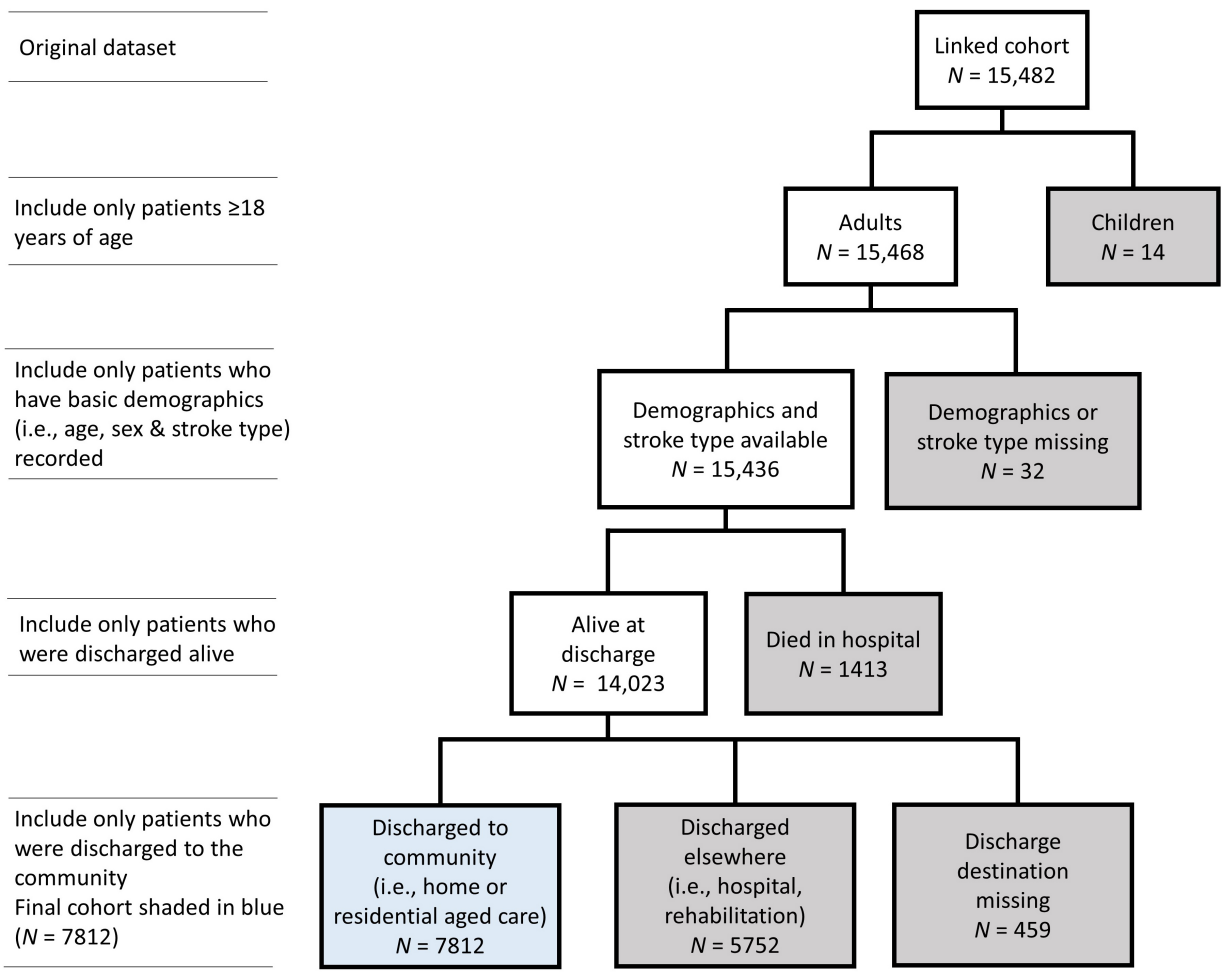
**Final cohort selection process**. Boxes shaded in grey indicate excluded registrants. TIA, Transient ischaemic attack.

Of 15,482 AuSCR registrants admitted between 2009–2013 (median age 76 years, 46% female, 64% ischaemic stroke), 7812 (50.4%) were eligible for this study (median age 73 years, 45% female, 57% ischaemic stroke; Fig. 1). Forty-seven percent (*N* = 3675) of eligible patients received a discharge care plan.

In univariable analyses, compared to patients who did not receive a care plan, those who did were more often younger, male, living in areas having socioeconomic advantage, or had a diagnosis of ischaemic stroke (Table 1). Similarly, patients who received a care plan at discharge were more often prescribed antihypertensive medications at hospital discharge, but were significantly less often transferred from another hospital, discharged on a weekend, or discharged to a residential aged care. Patients who did not receive a care plan were more often treated in a regional or large hospital than patients who did not receive a care plan. Length of hospital stay was greater among patients who received discharge planning (median 4 days), compared to those who did not (median 3 days), although this difference was not statistically significant. With the exception of dyslipidaemia, diabetes, and dementia, there was no significant difference in the prevalence of comorbidities between those who received discharge care plans and those who did not (Table 2).

**Table 1.** **Patient, clinical and system characteristics, overall and by receipt of a care plan at discharge**.

	Total cohort	Discharge care plan provided		*p*-value ^a^
		Yes	No	
	*N* = 7812	*N* = 3675	*N* = 4137	
	n (%)	n (%)	n (%)	
Patient characteristics				
Median age in years (Q1, Q3)	73.2 (62.6, 82.1)	72.9 (62.4, 81.6)	73.4 (62.7, 82.5)	0.014
Female	3489 (44.7)	1597 (43.5)	1892 (45.7)	0.043
Born in Australia	5068 (64.9)	2365 (64.4)	2703 (65.3)	0.363
Aboriginal or Torres Strait Islander	82 (1.1)	31 (0.85)	51 (1.2)	0.091
Interpreter required	300 (3.8)	147 (4.0)	153 (3.7)	0.489
Socioeconomic position ^b^				
Quintile 1 (Most disadvantaged)	1352 (17.3)	552 (15.0)	800 (19.3)	<0.001
Quintile 2	1195 (15.3)	549 (14.9)	646 (15.6)	
Quintile 3	1264 (16.2)	622 (16.9)	642 (15.5)	
Quintile 4	1540 (19.7)	737 (20.1)	803 (19.4)	
Quintile 5 (Least disadvantaged)	2461 (31.5)	1215 (33.1)	1246 (30.1)	
Clinical characteristics				
Type of stroke				
Ischaemic stroke	4446 (56.9)	2198 (59.8)	2248 (54.3)	<0.001
Intracerebral haemorrhage	560 (7.2)	259 (7.1)	301 (7.3)	
Transient ischaemic attack	2492 (31.9)	1094 (29.8)	1398 (33.8)	
Undetermined	314 (4.0)	124 (3.4)	190 (4.6)	
Unable to walk on admission ^c,d^	2721 (38.6)	1305 (38.4)	1416 (38.7)	0.735
Previous stroke	1522 (19.5)	738 (20.1)	784 (19.0)	0.208
In hospital stroke	202 (2.6)	97 (2.6)	105 (2.5)	0.778
Processes of care				
Treated in a stroke unit	5991 (76.7)	2795 (76.1)	3196 (77.3)	0.211
Transferred from another hospital	776 (9.9)	316 (8.6)	460 (11.1)	<0.001
Thrombolysis, if ischaemic	381 (4.9)	235 (6.4)	146 (3.5)	<0.001
Discharged on antihypertensives	5200 (66.6)	2827 (76.9)	2373 (57.4)	<0.001
Discharged on a weekend	919 (11.8)	320 (8.7)	599 (14.5)	<0.001
Discharged to aged care	844 (10.8)	323 (8.8)	521 (12.6)	<0.001
Length of stay ^e^				
Median length of stay in days (Q1, Q3)	4 (2, 6)	4 (2, 6)	3 (2, 6)	0.923
Short length of stay (<5 days)	3007 (39.4)	1430 (38.9)	1577 (39.9)	0.401
Hospital characteristics				
Regional hospital (*N* = 16 hospitals)	1472 (18.8)	526 (14.3)	946 (22.9)	<0.001
Teaching hospital (*N* = 10 hospitals)	3337 (42.7)	1586 (43.2)	1751 (42.3)	0.459
Large hospital (>300 beds; *N* = 22 hospitals)	6134 (78.5)	2836 (77.2)	3298 (79.7)	0.006

Q1, 25th percentile, Q3, 75th percentile. ^a^ *p*-value is based on chi-square test; ^b^ Defined using the Index of Relative Socioeconomic Advantage and Disadvantage; ^c^ Validated indicator of stroke severity; ^d^ 9.7% missing data; ^e^ 2.3% missing data.

**Table 2.** **Prevalence of comorbidities, overall and by receipt of a care plan at discharge**.

Comorbidities	Total cohort	Discharge care plan provided		*p*-value*^a^*
		Yes	No	
	*N* = 7812	*N* = 3675	*N* = 4137	
	n (%)	n (%)	n (%)	
Hypertension	4931 (63.1)	2330 (63.4)	2601 (62.9)	0.628
Atrial fibrillation	2001 (25.6)	959 (26.1)	1042 (25.2)	0.359
Angina	1381 (17.7)	622 (16.9)	759 (18.4)	0.100
Dyslipidaemia	1297 (16.6)	647 (17.6)	650 (15.7)	0.025
Carotid stenosis	470 (6.0)	223 (6.1)	247 (6.0)	0.856
Myocardial infarction	793 (10.2)	381 (10.4)	412 (10.0)	0.551
Congestive heart failure	744 (9.5)	348 (9.5)	396 (9.6)	0.877
Smoking	1710 (21.9)	835 (22.7)	875 (21.2)	0.094
Obesity	334 (4.3)	160 (4.4)	174 (4.2)	0.747
Diabetes	1310 (16.8)	658 (18.0)	652 (15.8)	0.011
Hemiplegia	2888 (37.0)	1370 (37.3)	1518 (36.7)	0.592
Liver disease	51 (0.7)	24 (0.7)	27 (0.7)	0.998
Cancer	780 (10.0)	376 (10.2)	404 (9.8)	0.493
Connective tissue disease	103 (1.3)	52 (1.4)	51 (1.2)	0.481
Human immunodeficiency virus	7 (0.1)	< $5^{b}$	< $5^{b}$	0.824
Peptic ulcer disease	180 (2.3)	86 (2.3)	94 (2.3)	0.842
Peripheral vascular disease	282 (3.6)	134 (3.7)	148 (3.6)	0.871
Chronic renal disease	745 (9.5)	348 (9.5)	397 (9.6)	0.849
Chronic pulmonary disease	600 (7.7)	297 (8.1)	303 (7.3)	0.210
Dementia	479 (6.1)	192 (5.2)	287 (6.9)	0.002
Overall comorbidity category				
None (CCI = 0)	4246 (54.4)	1964 (53.4)	2282 (55.2)	0.333
Moderate (CCI = 1)	1304 (16.7)	611 (16.7)	693 (16.8)	
Severe (CCI = 2)	879 (11.3)	430 (11.7)	449 (10.9)	
Very severe (CCI ≥3)	1383 (17.7)	670 (18.2)	713 (17.2)	

CCI, Charlson Comorbidity Index score. ^a^ *p*-value is based on chi-square test. ^b^ Cell sizes less than 5 are suppressed for confidentiality purposes.

In multivariable models, factors associated with a greater odds of receiving a discharge care plan included living in areas of greater socioeconomic position (OR 1.18, 95% CI 1.02–1.38), having a clinical diagnosis of ischaemic stroke (OR 1.18, 95% CI 1.05–1.33), being unable to walk on admission (i.e., more severe stroke, OR 1.15, 95% CI 1.01–1.31), and being discharged on antihypertensive medication (OR 3.07, 95% CI 2.69–3.50; Table 3). Similarly, the odds of receiving a discharge care plan increased over time (OR 1.39 per year, 95% CI 1.37–1.48). In contrast, factors associated with a reduced odds of receiving a care plan included being aged ≥85 years (*vs*<85 years; OR 0.79, 95% CI 0.64–0.96), being treated in a large hospital (OR 0.30, 95% CI 0.10–0.92), and being discharged on a weekend (OR 0.56, 95% CI 0.46–0.67) or to residential aged care (OR 0.48, 95% CI 0.39–0.60). Of all the comorbidities investigated, only a history of angina was associated with the odds of receiving a discharge care plan (OR 0.75, 95% CI 0.64–0.88). Having a greater CCI was not associated with receiving a discharge care plan.

**Table 3.** **Factors associated with receiving a care plan at discharge after stroke/TIA**.

Variables	Univariable	Multivariable	
	*N* = 7030 a	*N* = 7030 a	
	OR (95% CI)	OR (95% CI)	*p*-value
Age in years			
<65	Reference	Reference	
65–74	0.99 (0.87–1.12)	0.86 (0.73–1.01)	0.065
75–84	0.99 (0.87–1.12)	0.88 (0.74–1.03)	0.112
85+	0.85 (0.74–0.98)	0.79 (0.64–0.97)	0.021
Female	0.92 (0.83–1.01)	0.98 (0.87–1.10)	0.681
Year of stroke/TIA (per year)	1.26 (1.21–1.32)	1.39 (1.31–1.48)	<0.001
Aboriginal and Torres Strait Islander	0.76 (0.48–1.22)	0.89 (0.49–1.63)	0.711
Greater socioeconomic position ^b^	1.32 (1.19–1.46)	1.19 (1.02–1.38)	0.025
Ischaemic stroke	1.22 (1.11–1.34)	1.18 (1.05–1.33)	0.005
Unable to walk on admission ^c^	0.98 (0.89–1.08)	1.16 (1.02–1.32)	0.022
Comorbidities			
CCI (per score)	1.02 (0.99–1.04)	1.02 (0.98–1.05)	0.321
Angina	0.91 (0.80–1.03)	0.74 (0.64–0.88)	<0.001
Dyslipidaemia	1.16 (1.03–1.32)	1.06 (0.90–1.24)	0.489
Smoking	1.07 (0.96–1.20)	0.97 (0.84–1.12)	0.681
Diabetes	1.17 (1.03–1.33)	0.95 (0.79–1.13)	0.544
Dementia	0.76 (0.62–0.92)	1.07 (0.82–1.39)	0.620
Management in a large (300+ bed) hospital	0.90 (0.80–1.01)	0.30 (0.10–0.92)	0.035
Rural/regional hospital	0.57 (0.51–0.65)	0.32 (0.10–1.02)	0.054
Transferred from another hospital	0.77 (0.66–0.91)	1.16 (0.95–1.43)	0.143
Discharged on a weekend	0.61 (0.52–0.71)	0.56 (0.46–0.67)	<0.001
Discharged on antihypertensive agents	2.39 (2.15–2.65)	3.07 (2.69–3.50)	<0.001
Discharged to aged care	0.67 (0.57–0.78)	0.48 (0.39–0.60)	<0.001

OR, odds ratio obtained for a logistic regression model; CI, confidence interval; TIA, transient ischaemic attack; CCI, Charlson Comorbidity Index. ^a^ Analyses restricted to only participants with no missing data; ^b^ Quintiles 3–5 of the Index of Relative Socioeconomic Advantage and Disadvantage; ^c^ Validated indicator of stroke severity.

## 6. Discussion

In this study we identified patient-, process- and system-level factors associated with the provision of discharge care plan after stroke. In particular, our main finding that the day of discharge (weekend), discharge destination (residential aged care facility), and size of hospital (large, metropolitan) reduced the likelihood of receiving recommended discharge care plans was of concern. We also found that less than half of patients received a discharge care plan following stroke, indicating variations in the standard of management of stroke. Given the association of such variations with adverse long-term outcomes, such as death, poor quality of life, and more unmet needs [3, 29, 30], addressing the identified factors warrants urgent attention.

Reasons for preferential provision of care plans to patients with ischaemic stroke are currently unclear, and may be due to physician concern about bleeding resulting from these patients being discharged on single or dual antiplatelet therapy [31, 32]. Those who were prescribed antihypertensive medications at discharge in our cohort also had greater odds of receiving a discharge care plan. This indicates that hospitals which engage in routine discharge care planning for their patients are also more likely to adhere to other evidence-based practices for stroke, such as the provision of preventive medicines at discharge.

Patients aged ≥85 years are less likely to receive a care plan after acute stroke. Although all patients with stroke should receive guideline-recommended care irrespective of their age, there is evidence from an earlier study to suggest that being older is associated with sub-optimal provision of evidence-based care after stroke [33]. Cognitive decline in very elderly patients may underpin the clinician reasoning for not providing appropriate prevention management in older patients with stroke [34]. It is also possible that older patients are more likely to be receiving ongoing care from their regular healthcare practitioners, and may not require a discharge care plan. Nonetheless, discharge care planning should be tailored to the individual needs and goals of each patient and is considered to have significant benefit, and minimal harm for all patients, irrespective of age [5].

Patients who are discharged on a weekend less often receive a care plan at the time of hospital discharge than those discharged during the week, as do those discharged to an aged care facility [19]. Discharge processes for patients with stroke may be particularly sensitive to resourcing deficits on weekends, as care planning requires interactions between a diverse range of healthcare professionals from the multidisciplinary team. For those transitioning to residential aged care, there are particular challenges for patients and their families [35]. Barriers include, difficulties in coordinating a suitable time to meet with the patient, their family, and care staff, as previously reported [18]. Consequently, a reduced availability of the patient’s family while organising the transition to aged care may be a possible explanation as to why discharge care planning was not received. However, as we did not collect data on pre-stroke living arrangements, we could not explore whether there were any differences in the receipt of care plans between patients being discharged to an aged care facility for the first time and those returning to residential care after their stroke. We are unable to explain why large hospitals are less likely to provide a care plan at discharge. Further research is required to understand differences in the provision of discharge care planning based on hospital size.

A strength of this study was the use of a comprehensive linked dataset that allowed the investigation of many variables which would not have been possible for a study involving a single data source. Despite this, not all relevant factors were captured in the dataset, such as in-hospital complications, more sensitive measures of stroke severity (e.g., National Institutes of Health Stroke Scale), measures of pre- and post-stroke disability, and other pre-stroke variables such as living arrangements. Even though hospitals submit information to the AuSCR based on defined criteria, there is still an overall lack of standardisation for what is considered a “discharge care plan” (i.e., some hospitals may require different levels of documentation before this variable is considered to be a “yes”). Therefore, our results should be interpreted with caution in the context of these discrepancies. We acknowledge the limitations of using historical administrative data from 2009 to 2013, as they may not reflect the most contemporaneous practices for discharge care planning for acute stroke. However, adherence to discharge care planning is still sub-optimal based on more recent AuSCR and audit data [6, 7]. Our study is also limited by lack of data on other important factors, such as marital status, thrombolysis, discharge on antiplatelet or anticoagulant agents, and the presence of aphasia or dysarthria. Also, our measure of stroke severity, i.e., inability to walk on admission, may not reflect severity symptoms after treatment.

Several opportunities for future research exist in this area. Understanding clinical interpretations and application of current recommendations for discharge care planning will help to determine the underlying causes of inequitable discharge care (e.g., misinterpretation of the guidelines, need for clinician education, resource deficits), so that these areas can be targeted in future quality improvement projects. Whilst barriers and enablers of effective discharge care have been examined in the past, only staff from “high performing” hospitals with outstanding adherence to indicators of discharge care were interviewed [18]. In order to gain a more complete understanding of how the current discharge care planning recommendations are utilised, a variety of acute care staff from hospitals with varied adherence to indicators of discharge care should be recruited for future qualitative studies.

## 7. Conclusions

We have identified important factors that should be considered to improve discharge care planning processes after acute hospital care for stroke. Further research is needed to identify the best practices for delivering discharge care planning for all patients with stroke in the acute care environment. The most important aspect for future directions in this area will be to gather input from relevant stakeholders, such as patients and carers, clinicians, policy makers, and researchers. In this way, we can obtain different perspectives in order to optimise discharge care planning and ensure all patients with stroke are afforded this important process of care.

## Abbreviations

AuSCR, Australian Stroke Clinical Registry; ARIA+, Accessibility and Remoteness Index of Australia; CCI, Charlson Comorbidity Index; CI, Confidence Interval; IRSAD, Index of Relative Socioeconomic Advantage and Disadvantage; ICD-10-AM, International Statistical Classification of Diseases and Related Health Problems, Tenth Revision, Australian Modification; NHMRC, National Health and Medical Research Council; OR; Odds ratio; TIA, Transient ischaemic attack.

## Supplementary Material

Supplementary material associated with this article can be found, in the online version, at https://doi.org/10.31083/j.rcm2310328.
