# Supplementary material for: Factors Associated with Receiving a Discharge Care Plan After Stroke in Australia: A Linked Registry Study
Source: Rev Cardiovasc Med. 2022 Sep 28;23(10):328. doi: 10.31083/j.rcm2310328 (PMC11267321; doi:10.31083/j.rcm2310328)
Supplement: Supplementary file 1 [file 2153-8174-23-10-328-s1.docx]

**
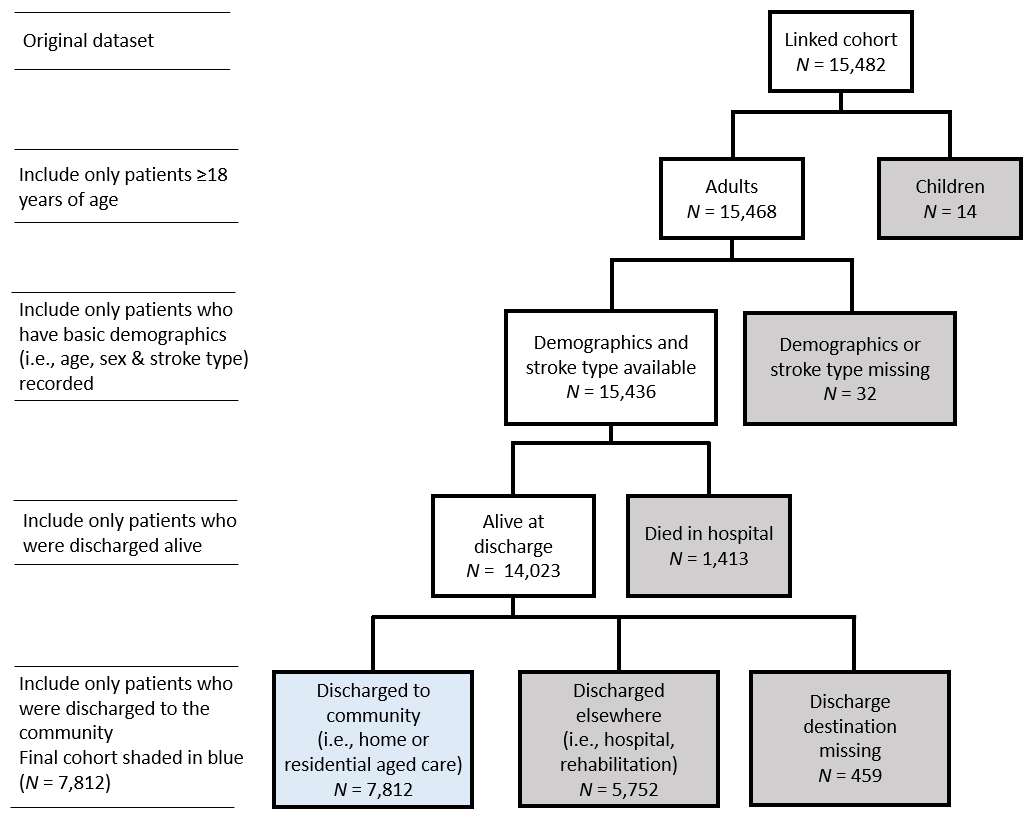
**

**Figure 1. Final cohort selection process.** *Boxes shaded in grey indicate excluded registrants. TIA, Transient ischaemic attack.*

**Supplemental Acknowledgements**

***Co-investigators and other contributors to the Australian Stroke Clinical Registry (does not include authors)***

The following people are acknowledged for their contribution to collecting hospital data on the patients registered in AuSCR or their participation on various governance committees:

**Steering and Management Committee**

**Geoffrey Donnan** MD (Stroke Division, The Florey Institute of Neuroscience and Mental Health VIC)

**Steering Committee**

**Julie Bernhardt** PhD (The Florey Institute of Neuroscience and Mental Health VIC); **Paul Bew** (The Prince Charles Hospital QLD); **Christopher Bladin** MD, MBBS, FRACP (Box Hill Hospital VIC, site investigator); **Greg Cadigan** BN (Queensland State-wide Stroke Clinical Network QLD);

**Helen Castley** MBBS (Royal Hobart Hospital Tasmania, site investigator); **David Dunbabin** MBBS FRACP FAFRM (Royal Hobart Hospital, TAS); **Anne Gordon** (The Royal Children’s Hospital Melbourne VIC); **Andrew Granger** (Osborne Park Hospital WA); **Niall Johnson** (Australian Commission on Safety and Quality in Health Care); **Erin Lalor** PhD (National Stroke Foundation VIC); **Andrew Lee** MBBS FRACP (Flinders Medical Centre, South Australia); **Richard Lindley** PhD (The George Institute for Global Health NSW); **Mark Mackay** MBBS, FRACP (Royal Children’s Hospital VIC, site investigator); **Sandra Martyn** (Health Statistics Centre Queensland Health QLD); **John McNeil** PhD (Monash University VIC); **Sandy Middleton**, PhD (Nursing Research Institute, St Vincent’s Health Australia NSW, Australian Catholic University NSW); **Michael Pollack** MBBS, FAFRM (RACP), FACRM, FFPM (ANZCA), MMedSci (Clin Epi) (Hunter Stroke Service NSW); **Peter Somerford** (Public Health Division of the WA Health Department WA); **Mark Simcocks** BSc (VIC, Consumer Representative); **Frances Simmonds** MSc(Med), (Australasian Rehabilitation Outcomes Centre NSW)

**Management Committee**

**Steven Faux** FAFRM (RACP) (St Vincent’s Health Australia NSW); **Kelvin Hill** BAppSci (Stroke Foundation VIC); **Christopher Price** (National Stroke Foundation)

**Site Investigators**

**Pradeep Bambery** MD, FRCP(G), FRACP (Bundaberg Hospital QLD); **Tim Bates** MBBS, FRACP (Swan District Hospital WA); **David Blacker** MBBS, FRACP (Sir Charles Gairdner Hospital WA); **Ernie Butler** MBBS FRACP (Peninsula Health VIC); **Sean Butler** FIMLS, BM Hons, MRCP(UK), FRACP (Prince Charles Hospital QLD); **Douglas Crompton** MA, PhD, MBBS, FRACP (Northern Hospital VIC); **Carolyn De Wytt** MRCP (UK), MB BCH DUBL, FRACP (Greenslopes Private Hospital QLD); **David Douglas** MBBS, M Admin, FRACGP, FAFRM (RACP) (Ipswich Hospital QLD); **Martin Dunlop** MBBS, FACRM (Cairns Base Hospital QLD); **Paula Easton** BPhty (Hons) (Mackay Hospital QLD); **Sharan Ermel** RN (Div1) (Bendigo Health VIC); **Nisal Gange** MBBS, AMC CERT (Toowoomba Hospital QLD); **Richard Geraghty** MBBS, FRACP (Redcliffe Hospital QLD); **Melissa Gill** BAppSc (SpPath) (Armidale Hospital, NSW); **Graham Hall** MBBS, FRACP (Princess Alexandra Hospital QLD); **Geoffrey Herkes** MBBS, PhD, FRACP (Royal North Shore Hospital NSW); **Karen Hines** BHIM (Caboolture Hospital QLD); **Francis Hishon** RN (Redland Hospital QLD); **James Hughes** BMed, FRACP (Tamworth Hospital NSW); **Joel Iedema** MBBS, FRACP (Redland Hospital QLD); **Martin Jude** MBBS, FRACP (Wagga Wagga Hospital NSW); **Paul Laird** MBBS, FRACP (Rockhampton Hospital QLD); **Graham Mahaffey** RN (Hervey Bay Hospital QLD); **Suzana Milosevic** MD, FRACP, AMC CERT (Logan Hospital QLD); **Peter O’Brien** MBBS, DIP RANZCOG, FRACMA, FACRRM (Warrnambool Hospital VIC); **Stephen Read** MBBS, PhD, FRACP (Royal Brisbane and Women’s Hospital QLD); **Fiona Ryan** BAppSc (SpPath), MHlthSc (Orange Hospital and Bathurst Hospitals NSW); **Arman Sabet** MD, FRACP, BSc (Gold Coast Hospital and Robina Hospital QLD); **Noel Saines** MBBS, FRACP (The Wesley Hospital QLD); **Eva Salud** MD, AMC CERT (Gympie Hospital QLD); **Amanda Siller** MBBS, FRACP (Queen Elizabeth II Jubilee Hospital QLD); **Christopher Staples** MD (Mater Adults QLD); **Richard White** MD, FRCP, FRACP. (Townsville Hospital QLD); **Andrew Wong** MBBS, PhD (Royal Brisbane and Women’s Hospital QLD)

**Staff at The Florey Institute of Neuroscience and Mental Health VIC**

Leonid Churilov, Alison Dias, Adele Gibbs, Brenda Grabsch, Francis Kung, Joyce Lim, Karen Moss, Sabrina Small, Renee Stojanovic, Steven Street, Emma Tod
